# Ontario Veterinary College

**Published:** 1882-04

**Authors:** 


### ONTARIO VETERINARY COLLEGE.

The annual examination of the students in attendance at the Ontario Veterinary College were brought to a close last Friday forenoon, and about one o'clock the presentation took place in the large lecture-room of the college. Dr. Smith, the principal, occupied the chair, and Mr. Duncan discharged the duties of secretary. Over one hundred students and graduates were present to take part in the proceedings, which were of the most enjoyable and enthusiastic character.

### PRIZEMEN.

The following is a list of the successful students to whom prizes were presented:

Pathology.—Silver medal, T. B. Cotton; 2d prize, W. A. Dryden; 3d, J. T. Claris.

Anatomy.—Silver medal, W. A. Dryden; 2d, J. Hugo Reed; 3d, Lyman Vandervoort.

Entoza.—Prize, D. P. Yonkerman.

Microscopy.—Prize, C. W. Stowe.

Physiology.—1st prize, J. Hugo Reed; 2d, D. P. Yonkerman.

Chemistry.—1st prize, J. Hugo Reed; 2d, D. P. Yonkerman.

For Anatomical Preparation.—Prize, W. Parkins.

Materia Medica.—J. Hugo Reed (senior), 1st prize.

Breeding and Management of Live Stock. –1st prize, $20, given by the Commissioner of Agriculture, J. Hugo Reed; 2d, $15, given by the Agricultural and Arts Association, T. B. Cotton, and $10 to J. N. Cook.

The gold medal, for the best general examination, was presented to Mr. J. Hugo Reed, of Georgetown, Ont.

GRADUATES.

The following is a list of the students who graduated this year:

W. S. Bell, Kars, Ont.; L. D. Blanchard, Mount Eaton, Ohio; H. G. Borneman, Clayton, Pa.; J. L. Brodie, New London, Iowa; G. W. Burt, Lynnvalley, Ont.; J. T. Claris, Buffalo, N. Y.; R. C. Clark, Wellesley Village, Ont.; J. N. Cook, Glanford, Ont.; S. J. Cottam, Edinburgh, Scotland; T. Bent Cotton, Mount Vernon, Ohio; W. A. Dryden, Tavistock, Ont.; F. Goulding, Richmond, Mich.; G. H. Hall, Chatham; J. Hodgins, London, Ont.; J. D. Johnston, Wahoo, Nebraska; J. Lawson, Acton, Ont.; W. G. Lyons, Cheltenham, Ont.; Alex. McDonald, Cobourg, Ont.; J. G. McInally, Lynnvalley; W. McLain, Nanticoke, Ont.; W. Parkins, Beeton; C. A. Pierce, Creston, Illinois; A. Porteous, Simcoe, Ont.; J. Price, Pine Lexington, Penn.; J. H. Reed, Georgetown, Ont.; W. T. C. Scanlon, London; C. L. Smith, Silver Cliff, Colorado; H. H. Sutherland, St. Francisville, Ill.; B. F. Swingly, Oregon, Ill.; A. Tanner, Drayton, Ont.; W. Tanner, Mount Forest, Ont.; F. A. Thomas, Paisley, Ont.; L. Vandervoort, Trenton, Ont.; A. A. Walker, Wingham, Ont.; J. A. Waugh, Pittsburg, Pa.; W. J. Waugh, Pittsburg, Pa.; A. E. Wessel, Wooler; J. Whytock, Teeswater; T. Wrigglesworth, Georgetown; D. P. Yonkerman, Cleveland, Ohio.

An address of a highly complimentary character was delivered by the Hon. Adam Crooks, who spoke of the excellent work which was being done by the college, in the success of which his colleagues of the Ontario Government took a deep interest. It afforded him the greatest pleasure to be called upon to take part in the presentation of prizes to the students. He resumed his seat amidst applause.

Addresses of a similar character were delivered by Prof. Buckland, Prof. Barratt, Prof. Thorburn, Mr. Wade, and other gentlemen, each of whom bore testimony to the good work which was being done by the college.

A life-size portrait was then presented to Dr. Smith by former graduates of the college, as a mark of esteem for him as Principal. It was accompanied by a complimentary address.

Principal Smith acknowledged the kindness of his friends in suitable terms, after which the proceedings were brought to a close.

